# Wnt5a Increases Properties of Lung Cancer Stem Cells and Resistance to Cisplatin through Activation of Wnt5a/PKC Signaling Pathway

**DOI:** 10.1155/2016/1690896

**Published:** 2016-11-08

**Authors:** Jiali Yang, Kangjian Zhang, Jing Wu, Juan Shi, Jing Xue, Jing Li, Juan Chen, Yongzhao Zhu, Jun Wei, Jinxi He, Xiaoming Liu

**Affiliations:** ^1^The Center of Laboratory Medicine, General Hospital of Ningxia Medical University, Yinchuan, Ningxia 750004, China; ^2^Department of Laboratory Medicine, College of Clinical Medicine, Ningxia Medical University, Yinchuan, Ningxia 750004, China; ^3^College of Life Science, Ningxia University, Yinchuan, Ningxia 750021, China; ^4^Department of Thoracic Surgery of General Hospital, Ningxia Medical University, Yinchuan, Ningxia 750004, China; ^5^Department of Pulmonary and Critical Care Medicine of General Hospital, Ningxia Medical University, Yinchuan 750004, China; ^6^Human Stem Cell Institute of General Hospital, Ningxia Medical University, Yinchuan, Ningxia 750004, China

## Abstract

The development of chemoresistance to cisplatin regimens causes a poor prognosis in patients with advanced NSCLC. The role of noncanonical Wnt signaling in the regulation of properties of lung cancer stem cells and chemoresistance* was interrogated*, by accessing capacities of cell proliferation, migration, invasion, and clonogenicity as well as the apoptosis in A549 cell lines and cisplatin-resistant A549 cells treated with Wnt5a conditional medium or protein kinase C (PKC) inhibitor GF109203X. Results showed that the noncanonical Wnt signaling ligand, Wnt5a, could promote the proliferation, migration, invasion, and colony formation in A549 lung adenocarcinoma cells and cisplatin-resistant A549/DDP cells and increase the fraction of ALDH-positive cell in A549/DDP cells. An exposure of cells to Wnt5a led to a significant reduction of A549/DDP cell apoptosis but not A549 cells. An addition of GF109203X could both strikingly increase the baseline apoptosis and resensitize the Wnt5a-inhibited cell apoptosis. Interestingly, an inhibition of Wnt/PKC signaling pathway could reduce properties of lung cancer stem cells, promote cell apoptosis, and resensitize cisplatin-resistant cells to cisplatin* via* a caspase/AIF-dependent pathway. These data thus suggested that the Wnt5a could promote lung cancer cell mobility and cisplatin-resistance through a Wnt/PKC signaling pathway and a blockage of this signaling may be an alternative therapeutic strategy for NSCLC patients with resistance to chemotherapies.

## 1. Introduction

Lung cancer is the leading cause of cancer-related death worldwide [1], of which the non-small-cell lung cancer (NSCLC) accounts for more than 80–85% of all patients with lung cancers [2, 3]. Despite huge progresses that have been made in treatments of this disease during last two decades, the prognosis and a five-year survival rate for patients with aggressive NSCLC remain poor [4, 5]. Cisplatin-based chemotherapy is the first-line therapy for advanced NSCLC, which is based on the formation of cisplatin-DNA that leads to DNA damage and sequentially activates apoptosis signaling pathways in cells [6]. However, the development of resistance to cisplatin leads to failure and poor prognosis in NSCLC patients treated with chemotherapy regimens. However, the mechanisms underlying chemoresistances are currently not fully understood; it is therefore a need to elucidate the molecular mechanism underpinning the cisplatin-resistance in order to develop effective therapeutic agents and/or strategies for NSCLC treatments.

Wnt pathways are developmental signaling that play fundamental roles in the regulation of various cell processes, including cell proliferation, survival, migration and polarity, and cell fate specification and stem cell self-renewal [7]. In addition to their roles in developments, tissue regeneration, and homeostasis, dysregulated Wnt signaling also contributes to the tumorigenesis and recurrence, as well as an enhanced potential of cancer stem cells (CSCs) and resistance to anticancer therapies in many types of cancers, including the lung cancer [8]. Based on the dependence of its key mediator *β*-catenin, the Wnt signaling pathway can be subdivided by a canonical (*β*-catenin-dependent) pathway and noncanonical (*β*-catenin-independent) signaling pathways [9]. The canonical Wnt/*β*-catenin signaling is one of the best characterized pathways, and the noncanonical Wnt pathways include at least the planar cell polarity (PCP) pathway and Wnt/calcium pathway [10]. In this context, the Wnt3a and Wnt5a are representative Wnt ligands that activate the canonical Wnt signaling and noncanonical Wnt signaling pathways, respectively [10]. In the Wnt/calcium pathway, the ligand binding of Wnt receptor/coreceptor(s) leads to an increased concentration of calcium through the protein kinase C (PKC); the increased intracellular calcium can also activate calcineurin and calmodulin-dependent protein kinase II (CaMKII). The calcineurin can interfere with TCF/*β*-catenin signaling in the Wnt/*β*-catenin pathway by activation of transforming growth factor beta-activated kinase 1 (TAK1) and nemo-like kinase (NLK). On the other hand, the CaMKII is able to induce activation of the transcription factor nuclear factor of activated T-cells (NFAT), which functionally regulates cell adhesion and migration [10].

Unlike the oncogenic roles of aberrant activation of Wnt/*β*-catenin signaling that has been well established in many cancers, the biological functions of noncanonical Wnt signaling in tumorigenesis are far to be characterized. In this regard, noncanonical Wnt signaling can be either a tumor suppressor or a tumor activator depending on cancer types [11]. For example, Wnt7a and Frizzled-9- (Fzd9-) mediated noncanonical Wnt signaling showed an antitumor activity [12–14], but Wnt5a exhibited contradictory effects on breast cancer [14]. However, more lines of study suggested that activation of noncanonical Wnt signaling, such as the Wnt5a-mediated signaling, was aberrantly enhanced in many epithelial cancers, including the lung cancer [15], breast cancer [16], ovarian cancer [17, 18], and gastric cancer [19]. With respect to Wnt5a signaling, it has been demonstrated to promote cancer cell migration and invasion [20], epithelial-mesenchymal transition (EMT) [18], and metastasis [17, 21, 22], as well as enhance the stemness of cancer stem cells (CSCs) and chemoresistances [21]. With respect to the chemoresistance, Wnt5a has been suggested as a biomarker of poor clinical outcome of patients with ovarian cancer and a mediator of chemoresistance [17]. Mechanistically, Wnt5a could regulate ATP-binding cassette, subfamily B (ABCB1, P-gp, and MDR1) expression in multidrug-resistant cancer cells through activation of the noncanonical protein kinase A (PKA)/*β*-catenin pathway [22]. In the lung, Wnt5a was found to correlate with cigarette smoke-related lung carcinogenesis by a mechanism of activating PKC/Akt pathway [23], by which Wnt5a inhibited lung cancer cell apoptosis [14]. Therefore, the noncanonical Wnt signaling, such as Wnt5a, has been suggested as potential therapeutic targets in cancers [24, 25].

In view of above findings, we thus hypothesized that Wnt5a-mediated noncanonical Wnt signaling pathways might play a regulatory role in chemoresistances of NSCLC cells. Results presented in this report showed that Wnt5a was able to increase the stemness of aldehyde dehydrogenase (ALDH) positive lung cancer stem cells with an enhanced capacity of cell proliferation, migration, invasion, and colony formation and inhibit cell apoptosis in both of lung adenocarcinoma A549 cells and cisplatin-resistant A549/DDP cells. Importantly, an inhibition of Wnt5a/calcium/PKC pathway by PKC inhibitor showed an increased cell apoptosis in cisplatin-resistant lung cancer A549/DDP cells. We thus identified a Wnt5a/PKC signaling pathway responsible for cisplatin-resistance in NSCLC cells.

## 2. Materials and Methods

### 2.1. Cell Lines and Wnt5a Conditioned Medium

Cell lines of human adenocarcinoma A549 (ATCC# CCL-185), the Wnt5a producing cell line, L Wnt5a (overexpressing mouse Wnt5a, ATCC# CRL-2814), and its control L cell line (ATCC# CRL-2648) were purchased from American Type Culture Collection (ATCC) (Manassas, VA, USA). The A549/DDP cell line, resistant to cisplatin, was purchased from the bank of cancer cell lines of Chinese Academy of Medical Science (Beijing, China) and its drug resistance phenotype was maintained in a medium contain 10 nM cisplatin (Cayman Chemical Company, Wilmington, DE, USA). PKC inhibitor (GF109203X) was purchased from Enzo Biochem (New York, USA). The final concentrations of both cisplatin and GF109203X were 2.5 *μ*M in this study. The cells were cultured and maintained at 37°C in a humidified atmosphere of 5% CO2 and 95% air in 1640 medium (Gibco, Grand Island, NY, USA) supplemented with 10% Fetal Bovine Serum (FBS) and 1% pen/strep. The Wnt5a and control L cells were grown to 80%–90% to be refreshed with 1640/5% FBS and cultured for additional 12 h. The culture media were then collected and used for preparation of Wnt5a-conditioned medium (Wnt5a-CM) and control medium (Control-CM), respectively [26]. Since transformed cell lines were used* in vitro* in this study, informed consent was not required, and there was not an ethnic concern either.

### 2.2. Cell Proliferation Assay

Cell proliferation was determined by using the Cell Counting Kit (CCK) (Bio-Rad Laboratories, Inc., Irvine, CA, USA). A549 or A549/DDP cells were split and seeded in a 96-well plate at a density of 2 × 10^4^ per well and treated with Wnt5a-CM, Wnt5a-CM plus GF109203X (PKC inhibitor), Control-CM, or Control-CM plus GF109203X for 12 h before they were treated with cisplatin for additional 24 h. The cells were then used for CCK assay.

### 2.3. Flow Cytometry Assay for Cell Apoptosis Analysis

Cells were cultured in different conditional media for 12 h prior to be treated with cisplatin for additional 24 h before they were used for analysis. For flow cytometry assay, cells were detached and labeled using an Annexin V-FITC/propidium iodide (PI) apoptosis detection kit (BD Pharmingen, USA) per manufacturer's instruction. Apoptotic and necrotic cells were quantified using a flow cytometer (BD FACSCalibur, San Jose, CA, USA) and the Cell Quest software. At least 10,000 cells were analyzed for each sample. Cells negative for Annexin V and PI were considered viable. Cells that were Annexin V+/PI− were indicative of early apoptosis, whereas Annexin V+/PI+ cells were considered as late apoptosis and necrotic cell populations.

### 2.4. Cell Scratch Assay

The scratch assay was used for accessing cell migration capacity. Cells were treated with different conditions for 12 h in 6-well culture plates. The cells were then scratched with 200 *μ*L pipette tips. The resultant unattached cells were removed by washing with prewarmed PBS for three times. Then the wounded monolayers cells were treated with distinct experimental conditions for additional 24 h. The closure of the wounded areas was observed under a microscope at 20x magnification (Leica, Germany) and photographed. The percentage of area of closure was quantified and calculated with the Pro Plus 6 (IPP6) image processing program. These experiments were performed in triplicate. Each condition was tested in duplicate and each experiment was repeated at least three times [27].

### 2.5. Transwell Assay

Invasion assay were performed transwell migration chambers (BD Biosciences, USA). The filters (8 *μ*m pore) were coated with 100 *μ*L Matrigel (BD Biosciences, USA), which was diluted to 1 : 2 concentration using serum-free 1640 medium, incubated at 37°C in a 5% CO2 atmosphere for 30 min for gelling. 2 × 10^4^ cells in 100 *μ*L of culture medium were seeded in the up chamber, and a 700 *μ*L conditional medium was added in the lower chamber. The conditional media included the Control-CM, Wnt5a-CM, Control-CM plus GF109203X, and Wnt5a-CM plus GF109203X. Cells were seeded at 2 × 10^4^ in 100 *μ*L media per upper-chambers, incubated at 37°C in a 5% CO2 atmosphere for 12 h. Remove medium and wash twice using precold PBS. Fix cells with 4% of paraformaldehyde for 20 min, and stain with 1% crystal violet for 20 min after the fixative was removed and the cells were washed with PBS twice [27].

### 2.6. Clonogenic Assay

A clonogenic assay was used for accessing the stemness of A549 and A549/DDP cells. For clonogenic analysis, single cell suspension of 1000 cells that treated with Wnt5a-CM or Control-CM with or without Wnt5a-CM plus PKC inhibitor (GF109203X) were seed on 6-well plate. Cells were continuously cultured for 10 days with refreshment of Wnt5a-CM or Wnt5a medium plus GF109203X with 3 days' interval. For colony counting, the medium was removed and the cells were rinsed with PBS prior to be fixed with 4% paraformaldehyde at room temperature for 5 min. After removing the fixation solution, the cells were then stained with 0.5% crystal violet solution and incubated at room temperature for 30 min. The staining solution was carefully removed, and the cells were rinsed with H2O to remove residual staining solution before air-drying the plate at room temperature for up to a day. The number of colonies from three 6-wells was counted and calculated under a light microscope and used as an index for clonogenicity. Each condition was tested in duplicate and each experiment was repeated for three times [27].

### 2.7. Immunoblotting (Western Blotting) Analysis

Whole cell extract was prepared by homogenizing the cells in a lysis buffer (50 mM Tris-HCl, pH 7.5, 5 mM EDTA, 150 mM NaCl, and 0.5% NP-40) for 60 min on ice. The lysates were then centrifuged at 12,000 ×g for 10 min at 4°C, and supernatants were collected as whole cell extracts. The cell extracts (70 *μ*g) were resolved in a 10% sodium dodecyl sulfate- (SDS-) polyacrylamide gel (SDS-PAGE) and before they were transferred to a PVDF membrane (Millipore, Billerica, MA, USA). The membrane was blocked in 4% fat free dry milk in PBS containing 0.2% Tween-20 and probed using rabbit anti-Wnt5a, Phospho-PKC, CaMKII (pan) antibody (Cell Signal Technology, USA), rabbit anti-Bcl2, caspase 3, BAX, beta-actin antibody (Proteintech, Wuhan, China), and rabbit anti-AIF (apoptosis inducing factor) (Boster, Wuhan, China). The blots were developed using the enhanced chemiluminescence (ECL) reagent (Advansta, Menlo Park, CA, United States) after they were incubated with the appropriate peroxidase labeled secondary antibodies. The levels of protein expression were semiquantified by optical densitometry using ImageJ Software version 1.46 (https://rsb.info.nih.gov/ij/). The ratio of the net intensity of each sample divided by the *β*-actin internal control was calculated as densitometric arbitrary units (AU) which was served as an index of relative expression of a protein of interest [28, 29].

### 2.8. ALDEFLUOR Assay

In order to access the fraction of aldehyde dehydrogenase (ALDH) positive cells, the ALDEFLUOR kit from Stemcell Technologies Inc. (Vancouver, Canada) was used per the manufacturer's protocol. In brief, cells treated with indicated conditions were placed in cytometry tubes containing the appropriate ALDEFLUOR buffer and the ALDH substrate, bodipy-aminoacetaldehyde (BAAA), and incubated for 45 min in darkness at 37°C. The enzyme inhibitor N,N-diethylaminobenzaldehyde (DEAB) was used as negative control, which results in the abolition of the fluorescent signal of ALDH-positive cells, and was used to compensate the flow cytometry signal (FACSCalibur, BD Biosciences). Flow cytometry profiling was performed on a FACScan flow cytometer (BD Biosciences). For the analysis of ALDH-positive cells, DEAB-treated sample was used as a negative control and ALDH activity in presence of DEAB was considered as a baseline. Data were analyzed using FlowJo software (Ashland, Covington, KY, USA).

### 2.9. Statistical Analysis

All data collected in this study was obtained from at least three independent experiments for each condition. SPSS17.0 analysis software (SPSS Inc., Chicago, IL, USA) and PRISM 5 (GraphPad software, La Jolla, CA, USA) were used for the statistic analysis. Statistical evaluation of the data was performed by one-way ANOVA when more than two groups were compared with a single control and* t*-test for comparison of differences between the two groups. Significant differences were assigned to *p* values <0.05 and <0.01 denoted by *∗* and *∗∗*, respectively. Data was presented as the mean ± standard deviation (SD).

## 3. Results

### 3.1. Wnt5a Enhances the Characteristics and Motility of Lung Cancer Cells

**Figure 1:**
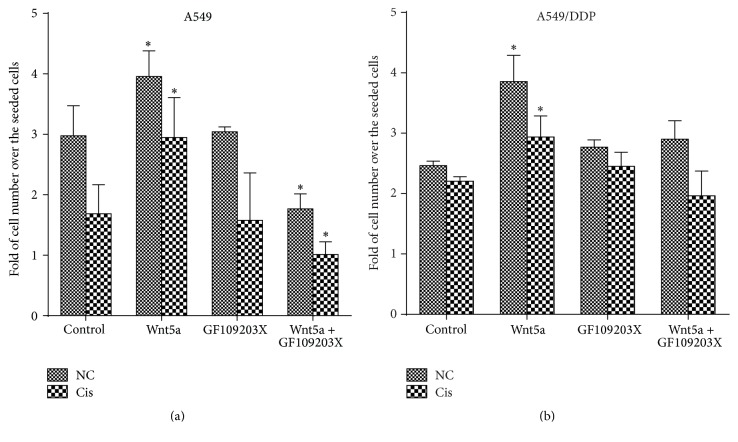
Wnt5a promotes lung cancer cell proliferation. Lung cancer cell line A549 and A549/DDP cells were cultured in 96-well plate at a density of 2 × 10^4^ per well and cultured in Wnt5a-CM and/or medium containing GF109203X. The proliferation of cells was then determined in terms of CCK assay. (a) The cell proliferation in A549 cells in the absence or presence of cisplatin. (b) The cell proliferation in A549/DDP cells in the absence or presence of cisplatin. Wnt5a showed an ability to promote cell proliferation, and GF109203X inhibited the Wnt5a-induced proliferation. Wnt5a-CM could increase cell proliferation in A549 and A549/DDP cells. Compared to a Control-CM treated control cells, *∗* represents *p* < 0.05. Data represented the mean ± SD from three independent triplicated experiments (*N* = 9). GF: GF109203X.

A compelling body of studies has revealed that Wnt5a is a regulator in the proliferation of normal and many cancer cell types [30, 31]. In order to access the potential impact of Wnt5a on metastatic properties of lung cancer cells, its effects on the proliferation, migration, and invasion in lung adenocarcinoma A549 cells and cisplatin-resistant A549/DDP cells were examined in terms of CCK assay for cell viability, scratch healing assay for cell migration, and transwell assay for cell invasion. As expected, both A549 cells and A549/DDP cells exposed to Wnt5a conditional medium (Wnt5a-CM) showed an increased cell proliferation in comparison with those cultured in the Control-CM, regardless of the presence of cisplatin (Figure 1). In contrast, an addition of Wnt/calcium/PKC signaling inhibitor GF109203X showed an inhibition of A549 cell proliferation (Figure 1(a)). Interestingly, the Wnt5a-induced cell proliferation could also be suppressed by an addition of GF109203X in A549 cells, which was also regardless of the presence of cisplatin (Figure 1(a)). However, the GF109203X had no effect on A549/DDP cell proliferation (Figure 1(b)). This result may suggest that the Wnt5a-mediated cell proliferation in A549 cells but not A549/DDP cells was through a Wnt5a/PKC pathway in A549 cells, implying that Wnt5a could induce lung cancer cell proliferation in a cell-context dependent manner.

**Figure 2:**
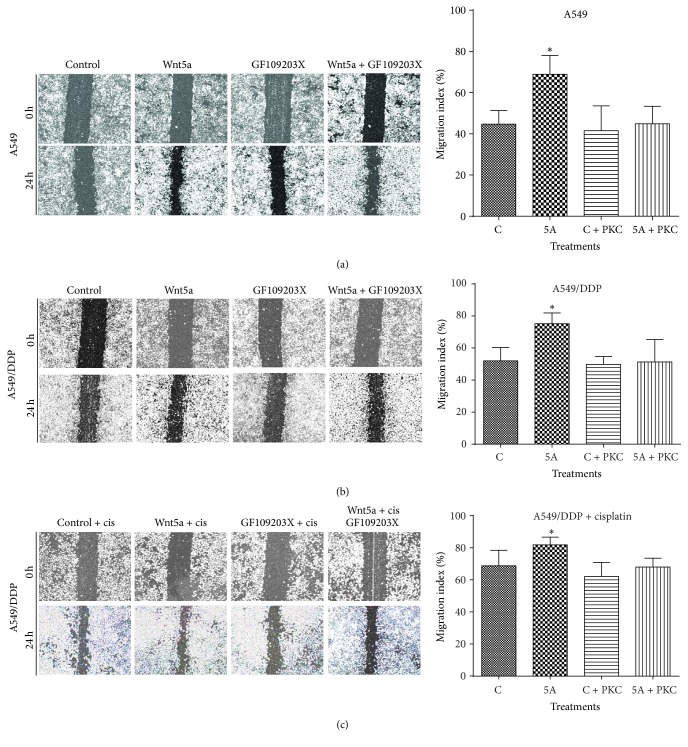
Wnt5a promotes cell migration in lung cancer cells* in vitro*. A549 and A549/DDP cells were cultured in Wnt5a-CM with or without PKC inhibitor GF109203X. The capability of cell migration was accessed in terms of a scratch assay. (a) Representative images of scratch assays for A549 cells (left panel) and its relevant quantification of the results of cell migration index (right panel). (b) Representative images of scratch assays for A549/DDP cells (left panel) and its relevant quantification of the results of cell migration index (right panel). (c) Representative images of scratch assays for A549/DDP cells in the presence of cisplatin (left panel) and its relevant quantification of the results of cell migration index (right panel). Wnt5a showed an ability to enhance cell migration in both A549 cells and A549/DDP cells. In contrast, GF109203X was able to inhibit the Wnt5a-promoted migration in these cells. Compared with Control-CM group, *∗* represents *p* < 0.05. Data represented the mean ± SD from three independent triplicated experiments (*N* = 9). GF: GF109203X.

**Figure 3:**
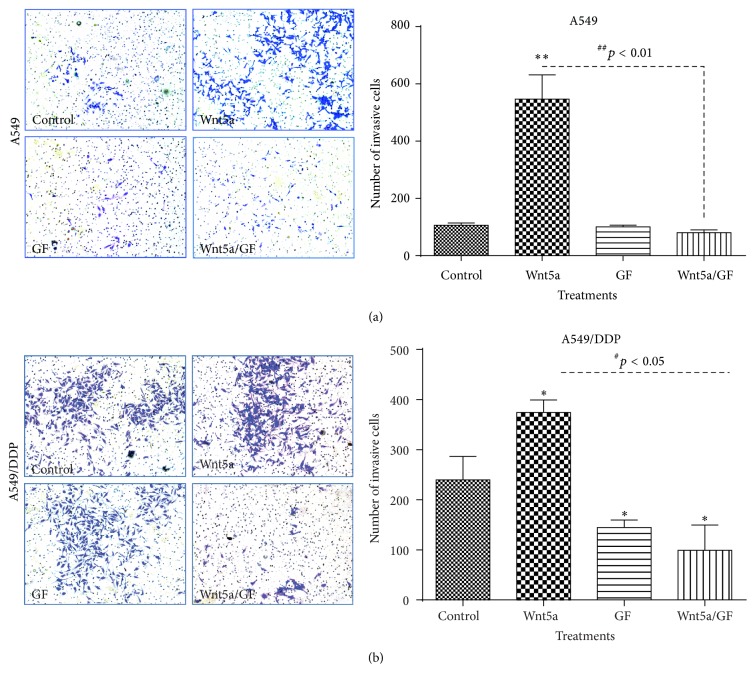
Wnt5a promotes cell invasion in lung cancer cells* in vitro*. A549 and A549/DDP cells were cultured with Wnt5a-CM with or without PKC inhibitor GF109203X in matrigel-coated transwells. The capability of cell invasion was accessed by a transwell assay. (a) Representative images of transwell assays for A549 cells (left panel) and its relevant quantification of the numbers of invasive cells (right panel). (b) Representative images of scratch assays for A549/DDP cells (left panel) and its relevant quantification of the numbers of invasive cells (right panel). Wnt5a showed an ability to significantly enhance cell invasion in both A549 and A549/DDP cells. In contrast, GF109203X was able to inhibit the Wnt5a-promoted invasion in these cells. Compared with Control-CM group, *∗* represents *p* < 0.05; *∗∗*: *p* < 0.01. Data represented the mean ± SD from three independent triplicated experiments (*N* = 9). GF: GF109203X.

Similarly inductive effects of Wnt5a were also observed in cell migration (Figure 2) and invasion (Figure 3) as determined by respective scratch and transwell assays, by which the Wnt5a showed abilities to promote cell migration (Figure 2) and invasion (Figure 3) in both A549 cells (Figures 2(a) and 3(a)) and A549/DPP cells (Figures 2(b), 2(c), and 3(b)) (*p* < 0.05). Of note, Wnt5a also exhibited a capacity to promote A549/DDP cell migration in the presence of cisplatin (Figure 2(c)) (*p* < 0.05). Consistently, PKC signaling inhibitor GF109203X alone had no effect on the migration in A549 and A549/DDP cells, but it could efficiently suppress the Wnt5a-mediated cell migrations of A549 and A549/DDP cells in all tested conditions (*p* < 0.01) (Figures 2(a), 2(b), and 2(c)). In addition, Wnt5a also exhibited an ability to promote cell invasion in these lung cells as observed in the transwell assay (*p* < 0.05) (Figure 3), and the addition of GF109203X inhibited the cell invasion in A549/DDP cells (Figure 3(b)) but not in A549 cells (Figure 3(a)), partially as a significant higher baseline invasive capacity in A549/DDP cell relative to the parent A549 cells (Figure 3 and data not shown). Equally noteworthy, the PKC inhibitor GF109203X could almost completely suppress the Wnt5a-induced cell invasion in A549 cells and A549/DDP cells (*p* < 0.05) (Figure 3).

### 3.2. Wnt5a Enhances the Clonogenic Capacity of Lung Cancer Cells

**Figure 4:**
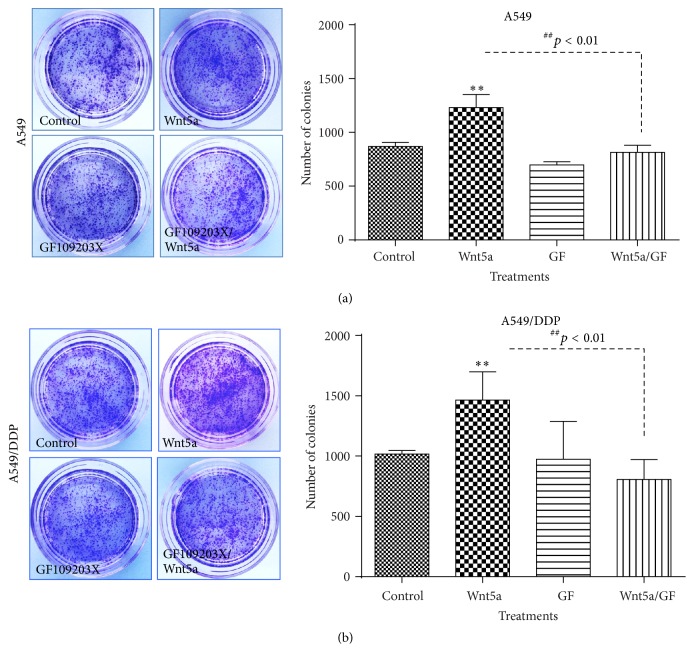
Wnt5a enhances the stemness of lung cancer cells determined by a clonogenic assay. A549 and A549/DDP cells were cultured with Wnt5a-CM with or without PKC inhibitor GF109203X; the capacity of clone formation was analyzed by using a clonogenic assay. (a) Representative images of clonogenic assay for A549 cells (left panel) and its relevant quantification of the total number of colonies from three 6-well plates (right panel). (b) Representative images of clonogenic assay for A549/DDP cells (left panel) and its relevant quantification of the total number of colonies from three 6-well plates (right panel). Wnt5a showed an ability to enhance clonogenic capacity in both A549 and A549/DDP cells. In contrast, GF109203X was able to inhibit the Wnt5a-enhanced clonogenicity in these cells. Compared with Control-CM group, *∗∗* represents *p* < 0.01. Data represented the mean ± SD from three independent triplicated experiments (*N* = 9). GF: GF109203X.

**Figure 5:**
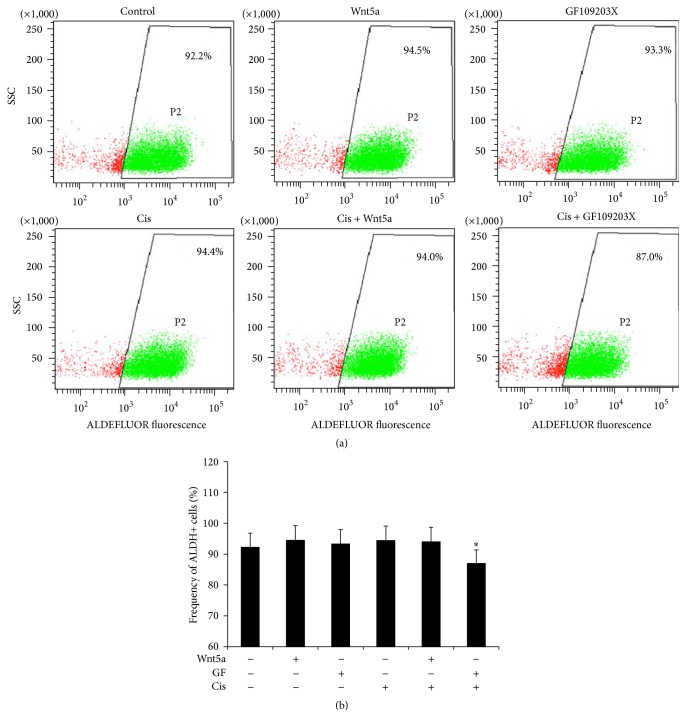
An inhibition of PKC signaling reduces the fraction of ALDH-positive in cisplatin-resistant lung cancer cells. A549/DDP cells were cultured in medium containing Wnt5a, PKC inhibitor GF109203X, and/or cisplatin for 24 hours; the cells were then harvested for ALDH staining and flow cytometric analysis. (a) Representative cytometric plots showed the positive fractions of ALDH-positive cells treated with indicated conditions. (b) A quantitative analysis of flow cytometry data showed Wnt5a slightly increased ALDH-positive cell fraction in A549/DDP cells in the absence of cisplatin, and an inhibition of PKC signaling by GF109203 significantly reduced the frequency of ALDH-cells in the presence of cisplatin. Compared to the corresponding non-GF109203X-treated group and other groups, *∗* represents *p* < 0.05. All data are represented as the mean ± SD of three independent triplicated experiments (*N* = 9). GF: GF109203X.

Colony forming ability is a hallmark of cancer stem cells (CSCs). Wnt signaling has been broadly recognized to play key roles in CSC self-renewal, proliferation, and differentiation [8, 32, 33]. In order to explore whether the Wnt5a-activated Wnt/PKC signaling has an effect on the characteristics of lung cancer cells, the colony forming capacity of A549 and A549/DDP cells was evaluated in terms of a clonogenic assay. An exposure of both A549 cells and A549/DDP cells to Wnt5a-CM resulted in a strikingly enhanced the capacity of colony formation in comparison with cells cultured in the Control-CM (*p* < 0.01) (Figure 4). In contrast, the Wnt5a-induced clonogenicity could be significantly inhibited by an addition of PKC inhibitor GF109203X in both A549 and A549/DDP cells (*p* < 0.01) (Figure 4), implying that the Wnt5a was able to enhance the potency of lung cancer stem cells by activation of Wnt5a/PKC pathway. In addition, ALDEFLUOR assay showed a high fraction (>90%) of ALDH-expressing cells in cisplatin-resistant cells, and cells cultured in Wnt5a-CM showed a slight increase of ALDH cell frequency but no statistical difference was observed as compared with Control-CM (Figure 5(a)). Of interest, an inhibition of PKC signaling by GF109203X led to a significantly reduced ALDH-positive fraction in A549-DDP cells (*p* < 0.05) (Figure 5(b)), implying that Wnt5a/PKC signaling may be involved in the stemness of lung cancer stem cells.

### 3.3. Wnt5a Activates PKC Signaling in Lung Cancer Cells

**Figure 6:**
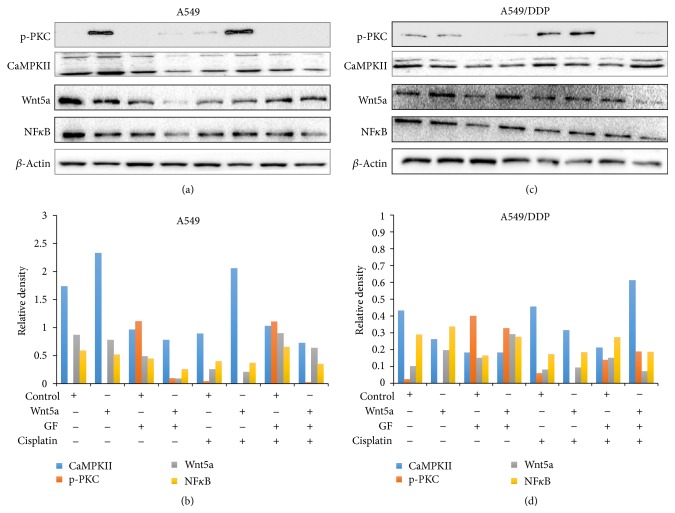
Wnt5a activates Wnt/calcium/PKC signaling in A549 and A549/DDP cells. A549 and A549/DDP were exposed to Wnt5a, PKC inhibitor GF109203X, and/or cisplatin during the culturing. The cells were then harvested for analyzing the expression of key components of Wnt/calcium/PKC signaling cascade. (a) Representative images of immunoblotting analysis in A549 cells. (b) The relative levels of proteins detected as evaluated by a densitometric analysis for proteins of interest of A549 cells in (a). (c) Representative images of immunoblotting analysis in A549/DDP cells. (d) The relative levels of proteins detected as evaluated by a densitometric analysis for proteins of interest of A549/DDP cells in (c). p-PKC: phosphorylated PKC.

 Several lines of evidence have suggested that the Wnt/PKC signaling pathway was aberrantly activated in some cancer types, in which Wnt5a might activate this signaling and was responsible for metastasis and chemoresistance in cancers in part through activation of Wnt/*β*-cateinin signaling [34–37]. Above* in vitro* studies have clearly showed that the PKC inhibitor GF109203X could sufficiently inhibit the Wnt5a-induced cell migration, invasion, and clonogenicity in both A549 and A549/DDP lung cancer cells, implying that Wnt5a could promote lung cancer cell mobility by activation of Wnt/PKC noncanonical pathway. The immunoblotting analysis revealed that Wnt5a could increase the expression of phosphorylated PCK (phos-PKC), CaMKII, and NF*κ*B in the Wnt/PKC signaling cascade, and PKC inhibitor GF109203X decreased the expression of phos-PKC, CaMKII, and NF*κ*B in both A549 (Figures 6(a) and 6(b)) and A549/DDP lung cancer cells (Figures 6(c) and 6(d)).

This result further confirmed that Wnt5a promote lung cancer cell metastasis by activation of Wnt/PCK signaling pathway.

### 3.4. Impacts of Wnt/PKC Signaling on the Apoptosis and Cisplatin-Resistance of Lung Cancer Cells

**Figure 7:**
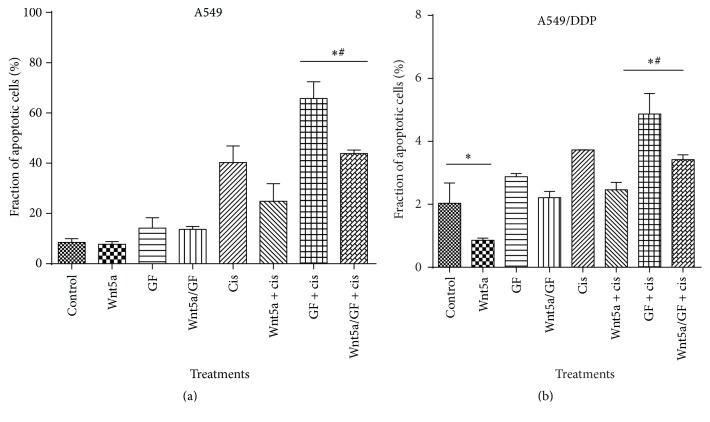
An inhibition of Wnt/calcium/PKC signaling induced apoptosis in lung cancer cells. A549 and A549/DDP cells were cultured in medium containing Wnt5a, PKC inhibitor GF109203X, and/or cisplatin. Cell apoptosis analyzed by a cytometric assay using an Annexin V-FITC/propidium iodide (PI) apoptosis detection kit. (a) Fractions of apoptotic cells in A549 cells treated with indicated conditions. (b) Fractions of apoptotic cells in A549/DDP cells treated with indicated conditions. An exposure of A549/DDP cells to Wnt5a showed a decreased cell apoptosis, but an inhibition of Wnt/calcium/PKC signaling by GF109203 increased the apoptosis in both A549 and A549/DDP cells, regardless of the presence of Wnt5a or cisplatin. Compared to the Control-CM group, *∗* represents *p* < 0.05. Compared to the corresponding non-GF109203X-treated group, # represents *p* < 0.05. All data are represented as the mean ± SD of three independent triplicated experiments (*N* = 9). GF: GF109203X.

**Figure 8:**
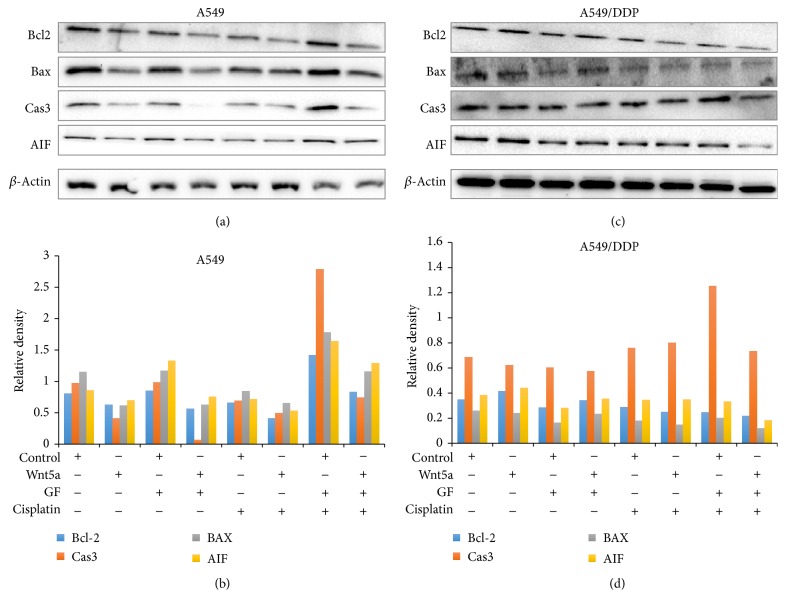
Apoptosis related proteins determined by an immunoblotting analysis. A549 and A549/DDP cells were cultured in medium containing Wnt5a, PKC inhibitor GF109203X, and/or cisplatin for 24 hours; the cells were then harvested for immunoblotting analysis of indicated proteins. (a) Representative images of immunoblotting analysis in A549 cells. (b) The relative levels of proteins detected as evaluated by a densitometric analysis for proteins of interest of A549 cells in (a). (c) Representative images of immunoblotting analysis in A549/DDP cells. (d) The relative levels of proteins detected as evaluated by a densitometric analysis for proteins of interest of A549/DDP cells in (c). An exposure of A549/DDP cells to Wnt5a reduced the expression of proapoptotic proteins (caspase 3, Bax, and AIF) but increased the expression of antiapoptotic protein Bcl-2 in lung cancer cells. Cas3: caspase 3.

Owing to Wnt5a being implicated in chemoresistance of several epithelial cancer cells, we next sought to investigate the influence of Wnt5a/PKC signaling in cell apoptosis and cisplatin-resistance in lung cancer cells. The result of flow cytometric analysis showed that Wnt5a and PCK inhibitor GF109203X had no effect on A549 cell apoptosis (Figure 7(a)). However, PCK inhibitor GF109203X could significantly promote cell apoptosis in A549 cells in the presence of cisplatin, and an exposure of cells to Wnt5a led to a significantly reduced cisplatin-induced cell apoptosis (*p* < 0.05) (Figure 7(a)). Interestingly, Wnt5a exhibited an inhibitory effect on the apoptosis of cisplatin-resistant A549/DDP cells, regardless of the presence of cisplatin (*p* < 0.05) (Figure 7(b)). An inhibition of Wnt/PKC signaling by GF109203X could significantly increase the cisplatin-induced cell apoptosis in A549/DDP cells, but only moderate increase of apoptotic cells was observed in cisplatin-resistant cells treated with PKC inhibitor without cisplatin (Figure 7(b)). Importantly, the PKC inhibitor-mediated increase of apoptosis could be diminished by the presence of Wnt5a-CM in A549/DDP cells (Figure 7(b)). Immunoblotting assay further identified that the Wnt5a-inhibited apoptosis and GF109203X-mediated increase of apoptosis were associated with a decreased and an increased expression of proapoptotic proteins of Bax, caspase 3, and apoptosis inducing factor (AIF) in lung cancer cells, respectively (Figure 8). These results were consistent with functions of Wnt5a reported in other human cancer types, including nasopharyngeal carcinoma (NPC) [21] and pancreatic cancer [30], which suggested that targeting Wnt/PKC signaling could resensitize cisplatin-resistant lung cancer cells to chemotherapy regimens in clinical settings.

## 4. Discussion

Involvements of Wnt signaling pathways, particularly the canonical Wnt pathway in the cancer development and progression, have been well established, in which Wnt/*β*-catenin signaling mainly plays an oncogenic role in cancer development, metastasis, and therapeutic resistances [7, 8, 14, 33, 39]. However, recent findings also suggested that noncanonical Wnt signaling pathways, such as a Wnt5a-mediated noncanonical signaling, might also serve as oncogene or tumor suppressor in a cancer cell type-dependent manner [40, 41]. In the lung cancer, an aberrant expression and/or alteration of the Wnt signaling components also are observed in 50% of human NSCLC cell lines and lung cancer resected samples [42], in which Wnt signaling is associated with increased proliferation and metastatic properties of lung cancer cells, poor prognosis, and resistances to conventional chemoradiotherapies and targeted therapies in lung cancer patients [14, 33, 43–45]. Of interest, implications of dysregulated noncanonical Wnt signaling and the cancer type-dependent oncogenic or tumor suppressor role of Wnt signaling in tumorigenesis have recently spurred an increasing attention in the metastasis and therapeutic resistances of lung cancer [14, 22, 23, 25, 31, 36, 45–47].

In the present study, the role of the Wnt5a-mediated noncanonical signaling in regulation of characteristics, stemness, and cisplatin-resistance in lung adenocarcinoma A549 and cisplatin-resistant A549 cells was examined* in vitro*. The results demonstrated that Wnt5a could enhance the capacity of proliferation, migration, invasion, and colony formation but reduce cell apoptosis in lung cancer cells, through activation of Wnt/calcium/PKC signaling pathway. Importantly, the PKC inhibitor GF109203X could reduce the fraction of ALDH-positive lung cancer stem cells in A549/DPP cells and reverse the Wnt5a-inhibited cell apoptosis in A549 cells. Moreover, the GF109203X significantly induced the Wnt5a-inhibited apoptosis in cisplatin-resistant cell at least in part* via *a caspase/AIF-dependent pathway. These results were in agreement with a study by Stewart [14], in which the author also found that multiple Wnt ligands, receptors, and other key components, including Wnt-1, Wnt-2, Wnt-3, and Wnt-5a, and those of Wnt pathway components Frizzled-8, Dishevelled, porcupine, and TCF-4 were elevated in NSCLC tissues [14]. Such an activated Wnt signaling was associated with poor prognosis in NSCLC. Conversely, they also found, Wnt-7a, another noncanonical ligand that played a tumor suppressor role in NSCLC, which could suppress NSCLC development and was often downregulated in this type of cancer [14]. Together with our findings, these studies suggested complex roles of Wnt signaling, particularly the noncanonical Wnt signaling in NSCLC. These data thus also suggest that the Wnt/PKC signaling pathway may be a novel target for developing a therapeutic intervention to target lung CSCs and reverse the cisplatin-resistance in lung cancer cells [17, 18, 21, 23, 25, 46–48].

Aberrant activation of both canonical and noncanonical Wnt signaling has been shown to promote tumorigenesis in lung cancer, particularly in the NSCLCs [14, 49]. Although a noncanonical Wnt signaling was found to suppress the canonical pathway in certain types of NSCLC such as squamous cell carcinoma, such a downregulated canonical Wnt signaling was not correlated with improved prognosis but led to a decreased cell adhesion and an increased EMT potential [43]. These results suggest that the noncanonical Wnt signaling is able to increase metastasis and serves as a key potential biomarker for diagnosis and prognosis, as well as a therapeutic target for developing novel and effective treatments in lung cancers [14, 23, 45, 50]. In this regard, the Wnt5a is a representative ligand that activates noncanonical Wnt signaling in regulation of cell migration and polarity during embryonic morphogenesis, which is normally downregulated in adult tissues. However, a constitutive expression or increased expression of Wnt5a and its receptor Ror2 is involved in enhanced invasive and metastatic properties of many cancer types, suggesting an oncogenic role of Wnt5a/Ror2 signaling in tumorigenesis [17, 21, 40, 51–54]. In addition to its oncogenic roles in cancers, Wnt5a also has been found to inhibit canonical Wnt/*β*-catenin signaling in a Ror2-dependent manner and has a tumor suppressor role in several cancer types, such as colorectal cancer (CRC) [55], thyroid cancer [56], and maybe breast cancers as well [34, 36, 57]. With respect to its antitumor activity, Wnt5a is able to inhibit tumor invasion and metastasis by antagonizing the Wnt/*β*-catenin signaling pathway and inducing the degradation of *β*-catenin [32]. For instance, Wnt5a was frequently silenced due to promoter methylation in CRCs. An enforced expression of Wnt5a led to a degradation of *β*-catenin and a reduced expression of Cyclin D1 and capacity of colony formation, indicating that Wnt5a acted as a tumor suppressor role in CRC by suppressing the canonical Wnt signaling pathway [55]. However, contradictory effects of Wnt5a have been reported in breast cancer, in which a loss of Wnt5a expression was associated with high-histological grade, high-mitotic index, and negative expression of the estrogen and progesterone receptors in the invasive ductal breast cancer [57]. However, another report identified that Wnt5a could induce invasiveness of cancer cells and the production of matrix metalloproteinase-7 (MMP7) and tumor necrosis factor-*α* (TNF-*α*) in macrophages, which was essential for macrophage-induced invasiveness in breast cancer [58]. Nevertheless, more lines of evidence indicated that activation of Wnt5a-mediated signaling was associated with malignant capacity of many cancer types, which exhibited paradoxical effects on tumorigenicity, resistance to therapeutic agents, and/or metastasis in a cancer cell type-dependent manner [47, 51, 53]. In the case of lung cancer, Wnt5a exhibited an ability to promote cell migration and invasion* in vitro* and metastasis* in vivo*, which was significantly associated with malignant features of many cancer types [23, 51, 53, 59]. Consistently with previous findings, we also demonstrated that Wnt5a could enhance the mobility and stemness of lung cancer cells, including migration, invasion, colony formation, and chemoresistance, through a Wnt/PKC pathway.

Indeed, an early microarray analysis for gene expression profiling demonstrated that Wnt5a could directly promote melanoma cell invasion and motility* via* inducing expression of Slug, which in turn promotes EMT gene expression in a PKC-dependent manner [60, 61]. Such a Wnt5a/PKC-promoted EMT was also found in epithelial ovarian cancer [18] and oral squamous cell carcinoma (OSCC) [20]. In the epithelial ovarian cancer, an increased expression of Wnt5a and PKC*α* was significantly correlated with metastasis of the disease, and the PKC*α* inhibitor could reduce the metastatic capacity [18]; in OSCC, Wnt5a was found to promote the migration and invasion of cancer cells through activation of noncanonical Wnt/Calcium/PKC pathway [20]. In addition, a recent high-throughput gene expression profiling further revealed that Wnt5a signaling was upregulated in highly metastatic nasopharyngeal carcinoma (NPC) cells and tissues, in which Wnt5a was able to activate PKC signaling and form a positive Wnt5a and phosphorylated PKC loop to promote the stemness characteristics of NPC cells, leading to an enhanced metastatic features and tumorigenicity* in vitro* and* in vivo* [21]. In lung cancer, Whang et al. also found that exposure of human bronchial epithelia (HBE) cells to cigarette smoke condensate showed an activated Wnt5a/PKC/Akt signaling and increased cell proliferation and clonogenicity. A higher expression of Wnt5a was also found in tumor tissues of smokers relative to matched normal tissues. A blockage of Wnt5a/PKC led to a significantly decreased cell viability and clonogenicity, along with an increased apoptosis* via* the downregulation of Bcl2 and induction of cleaved poly-ADP-ribose polymerase [23]. In addition, Wnt5a was transcribed based on multiple mechanisms, including Notch, Hedgehog, TGF*β*, and NF-*κ*B signaling cascades [62]. In agreement with these findings, our results also suggested that a Wnt5a/PKC-mediated caspase-dependent apoptosis signaling pathway and the NF-*κ*B signaling cascade were involved in the metastatic potentials, stemness, and chemoresistance in A549 and cisplatin-resistant A549/DDP NSCLC cells. Of importance, an inhibition of PKC signaling by GF109203X led to a significantly decreased fraction of ALDH-expressing cells in A549/DDP cells in the presence of cisplatin. The ALDH has been recognized as a cancer stem cell marker in several cancer types, including the lung cancer [63–68]. This finding implies that targeting of PKC pathway may reverse chemoresistance in part by reducing stemness of CSC in lung cancer cells.

Apart from the oncogenic role of Wnt signaling in cancer metastasis and invasion, dysregulations of Wnt signaling pathways, particularly the canonical Wnt pathway, have been demonstrated to contribute to therapeutic resistances in many cancer types [31, 37, 44, 69–72]. In this regard, Wnt5a was also found to modulate cell cycle progression and contributes to the chemoresistance in pancreatic cancer cells [70] and regulates ABCB1 expression in multidrug-resistant breast cancer cells through activation of the noncanonical PKA/beta-catenin pathway [48]. In the NSCLC cells, activation of Wnt pathway was previously demonstrated to be associated with resistance to platinum-based chemotherapy [37, 50, 73]. Cisplatin-resistant NSCLC cells had increased expression of Wnt pathway genes. An exposure of these cells to a Wnt inhibitor led to an inhibited cell proliferation and sensitized NSCLC cells to chemotherapeutic agent docetaxel [74] and cisplatin [75], along with a downregulated expression of Wnt signaling target genes. In this study, we showed that a blockage of Wnt/PKC signaling could increase the baseline apoptosis and cisplatin-induced apoptosis in NSCLC A549 cells and cisplatin-resistant A549/DDP cells, suggesting that targeting Wnt/PKC noncanonical Wnt signaling may be a potential therapeutic strategy to circumvent chemoresistance in lung cancer treatment.

## 5. Conclusion

**Figure 9:**
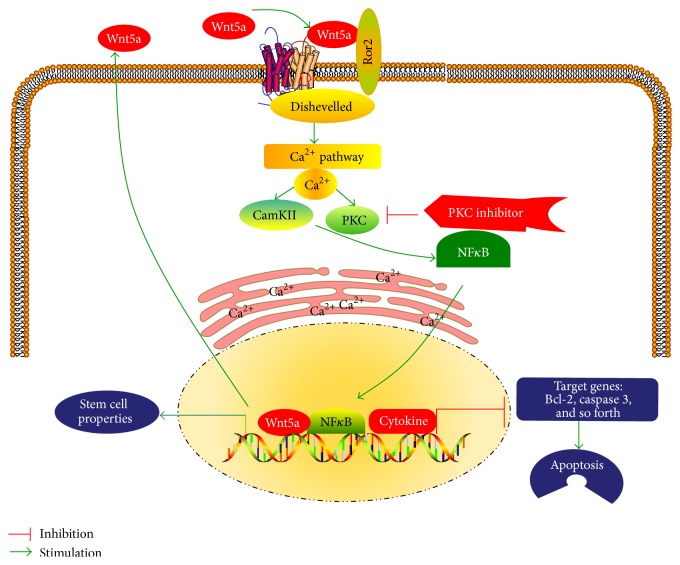
A schematic model to summarize Wnt5/PKC signaling pathway in lung cancer. Wnt5a activates PKC signaling and leads to endoplasmic reticulum (ER) release of Ca^2^+; PKC and CaMKII are activated, which in turn activate NF*κ*B. Subsequently, the activated NF*κ*B signaling promotes stem cell properties and inhibits apoptosis in lung cancer. CaMK: calcium/calmodulin kinase; FZD: Frizzled; PKC: protein kinase C; Ror: receptor tyrosine kinase-like orphan receptor.

In conclusion, in the present study, we examined the potential of Wnt5a-mediated noncanonical Wnt signaling in the mobility and chemoresistance of NSCLC cells and stemness of lung CSCs. The results demonstrated that Wnt5a could promote proliferation, migration, invasion, and colony formation, as well as inhibit cell apoptosis in both A549 and cisplatin-resistant A549/DDP NSCLC cells. Moreover, a blockage of Wnt5a signaling by PKC inhibitor GF109203X could strikingly reduce the mobility and increase both the baseline apoptosis and the cisplatin-induced apoptosis in A549 and A549/DDP cells and reverse the inhibitory function of Wnt5a in cell apoptosis. Mechanistically, the Wnt5a activated Wnt5a/PKC signaling cascade and led to activation of endoplasmic reticulum (ER) release of Ca^2+^, PKC, and CaMKII, which in turn activated NF*κ*B, subsequently promoted stemness, and inhibited apoptosis in NSCLC cells (Figure 9). This study thus suggests that Wnt5a/PKC signaling may play an important role in the metastasis, chemoresistance, and stemness of lung CSCs, which may provide a novel clue for understanding the crucial role of noncanonical Wnt signaling in lung carcinogenesis and therapeutic resistances.

## Abbreviations

ABCB: ATP-binding cassette, subfamily B
ALDH: Aldehyde dehydrogenase
BAAA: Bodipy-aminoacetaldehyde
CaMKII: Calmodulin-dependent protein kinase II
CSC: Cancer stem cell
DEAB: N,N-Diethylaminobenzaldehyde
EMT: Epithelial-mesenchymal transition
NLK: Nemo-like kinase
NFAT: Nuclear factor of activated T-cells
NSCLC: Non-small-cell lung cancer
PCP: Planar cell polarity
PKC: Protein kinase C
TAK1: Transforming growth factor beta-activated kinase 1.
